# Clinical Characteristics and Prognosis of Heart Failure with Preserved Ejection Fraction Across Diverse Ejection Fraction Ranges

**DOI:** 10.31083/j.rcm2505177

**Published:** 2024-05-20

**Authors:** Jingjing Su, Kangkang Su, Yanping Song, Lihui Hao, Yitao Wang, Shuxia Chen, Jian Gu

**Affiliations:** ^1^School of Medicine, Graduate School of Hebei Medical University, 050017 Shijiazhuang, Hebei, China; ^2^Department of Heart Center, Hebei General Hospital, 050051 Shijiazhuang, Hebei, China; ^3^School of Medicine, Graduate School of Hebei North University, 075000 Zhangjiakou, Hebei, China; ^4^School of Medicine, North China University of Science and Technology, 063210 Tangshan, Hebei, China

**Keywords:** chronic heart failure, cardiovascular disease, left ventricular ejection fraction, prognosis

## Abstract

**Background:**

Recent studies have indicated that heart failure (HF) with preserved ejection fraction (HFpEF) within different left ventricular ejection fraction (LVEF) ranges presents distinct morphological and pathophysiological characteristics, potentially leading to diverse prognoses.

**Methods:**

We included chronic HF patients hospitalized in the Department of Cardiology at Hebei General Hospital from January 2018 to June 2021. Patients were categorized into four groups based on LVEF: HF with reduced ejection fraction (HFrEF, LVEF ≤40%), HF with mildly reduced ejection fraction (HFmrEF, 41% ≤ LVEF ≤ 49%), low LVEF-HFpEF (50% ≤ LVEF ≤ 60%), and high LVEF-HFpEF (LVEF >60%). Kaplan‒Meier curves were plotted to observe the occurrence rate of endpoint events (all-cause mortality and cardiovascular mortality) within a 2-year period. Cox proportional hazards regression models were employed to predict the risk factors for endpoint events. Sensitivity analyses were conducted using propensity score matching (PSM), and Fine-Gray tests were used to evaluate competitive risk.

**Results:**

A total of 483 chronic HF patients were ultimately included. Kaplan‒Meier curves indicated a lower risk of endpoint events in the high LVEF-HFpEF group than in the low LVEF-HFpEF group. After PSM, there were still statistically significant differences in endpoint events between the two groups (all-cause mortality *p* = 0.048, cardiovascular mortality *p* = 0.027). Body mass index (BMI), coronary artery disease, cerebrovascular disease, hyperlipidemia, hypoalbuminemia, and diuretic use were identified as independent risk factors for all-cause mortality in the low LVEF-HFpEF group (*p* < 0.05). Hyperlipidemia, the estimated glomerular filtration rate (eGFR), and β-blocker use were independent risk factors for cardiovascular mortality (*p* < 0.05). In the high LVEF-HFpEF group, multivariate Cox regression analysis revealed that age, smoking history, hypoalbuminemia, and the eGFR were independent risk factors for all-cause mortality, while age, heart rate, blood potassium level, and the eGFR were independent risk factors for cardiovascular mortality (*p* < 0.05). After controlling for competitive risk, cardiovascular mortality risk remained higher in the low LVEF-HFpEF group than in the high LVEF-HFpEF group (Fine-Gray *p* < 0.01).

**Conclusions:**

Low LVEF-HFpEF and high LVEF-HFpEF represent two distinct phenotypes of HFpEF. Patients with high LVEF-HFpEF have lower risks of both all-cause mortality and cardiovascular mortality than those with low LVEF-HFpEF. The therapeutic reduction in blood volume may not be the best treatment option for patients with high LVEF-HFpEF.

## 1. Introduction

Heart failure (HF) is the ultimate stage of all heart diseases, affecting approximately 40 million individuals globally, with its incidence and mortality rates increasing annually [1, 2]. HF with preserved ejection fraction (HFpEF) is a subtype of HF, accounting for approximately half of all HF cases, with a 5-year survival rate of only approximately 50% [3, 4]. HFpEF has long been considered an independent subtype of HF. However, recent research has indicated that the pathophysiological mechanisms of HFpEF vary across different ranges of left ventricular ejection fraction (LVEF). Additionally, morphological and functional differences exist between them [5], which may result in distinct prognoses. Therefore, the purpose of this study was to evaluate the clinical characteristics and outcomes of HFpEF patients with different LVEF ranges to provide a basis for better individualized treatment approaches for HF patients.

## 2. Data and Methods

### 2.1 Subjects of the Study

This study is a single-center case‒control study that included hospitalized patients with chronic HF at Hebei General Hospital from January 2018 to June 2021. Inclusion criteria were as follows: (1) diagnosis of HF meeting the diagnostic criteria outlined in the “2022 American Heart Association/American College of Cardiology/Heart Failure Society of America (AHA/ACC/HFSA) Guideline for the Management of Heart Failure” [6]; (2) age ≥18 years; and (3) initial echocardiography performed within 24 hours of admission and repeat echocardiography conducted 3–6 months after discharge. Exclusion criteria were as follows: (1) lack of echocardiography or having only one echocardiography examination; (2) mental or behavioral disorders and inability to cooperate with follow-up; (3) recent severe infections; (4) missing clinical data; and (5) severe liver or kidney dysfunction, malignant tumors, or other conditions significantly threatening the patient’s short-term survival.

This study was reviewed and approved by the Ethics Committee of Hebei General Hospital, with informed consent waived (NO.2023142).

### 2.2 Data Collection and Grouping

Baseline information of chronic HF patients, including age, sex, smoking history, physical examination, comorbidities, treatment details, laboratory tests, and cardiac ultrasound results, was obtained from the electronic medical record system. Patients with baseline and follow-up LVEF ≥50% were categorized as the HFpEF group. Patients with baseline LVEF >40% and follow-up LVEF in the range of 41% to 49% were classified as having HF with mildly reduced ejection fraction (HFmrEF). Regardless of baseline LVEF, patients with follow-up LVEF ≤40% were defined as having HF with reduced ejection fraction (HFrEF). Among HFpEF patients, they were further divided into the low LVEF-HFpEF group (50% ≤ LVEF ≤ 60%) and the high LVEF-HFpEF group (LVEF >60%) based on their follow-up LVEF. LVEF was calculated by measuring left ventricular end-diastolic and end-systolic volumes using a modified Simpson method based on two-dimensional echocardiography.

### 2.3 Follow-up and Endpoint Events

To observe the survival status of patients within 2 years, all patients were followed up through outpatient visits, telephone interviews, and medical records, with data collection on endpoint events and their respective timeframes. The follow-up period extended until June 1, 2023.

### 2.4 Statistical Analysis

Statistical analysis was performed using SPSS 26.0 (SPSS Inc., Chicago, IL, USA) and R software version 4.1.1 (R Foundation for Statistical Computing, Vienna, Austria). Normally distributed or approximately normally distributed continuous data are presented as the mean ± standard deviation and were compared between groups using independent-sample *t *tests when homogeneity of variance was met or Dunnett’s *T3* test when variance was not homogeneous. Highly skewed continuous data are presented as M (*$P_{25}$*, *$P_{75}$*), and group comparisons were conducted using the Kruskal‒Wallis test followed by pairwise comparisons using the Mann‒Whitney *U* test with Bonferroni correction for multiple comparisons. Categorical data were expressed as rates or percentages, and group comparisons were made using the chi-square test or Fisher’s exact test with Bonferroni correction for multiple comparisons. Kaplan‒Meier curves were constructed for overall mortality and cardiovascular mortality, and comparisons were made using the log-rank test. Cox proportional hazards regression models were used to assess risk factors for endpoint events. Sensitivity analysis was conducted using 1:1 propensity score matching (PSM) and nearest neighbor matching. The Fine-Gray test was employed to assess competing risks between the high LVEF-HFpEF and low LVEF-HFpEF groups. Statistical significance was set at α = 0.05.

## 3. Results

### 3.1 General Clinical Characteristics of Enrolled Patients

**Fig. 1.**
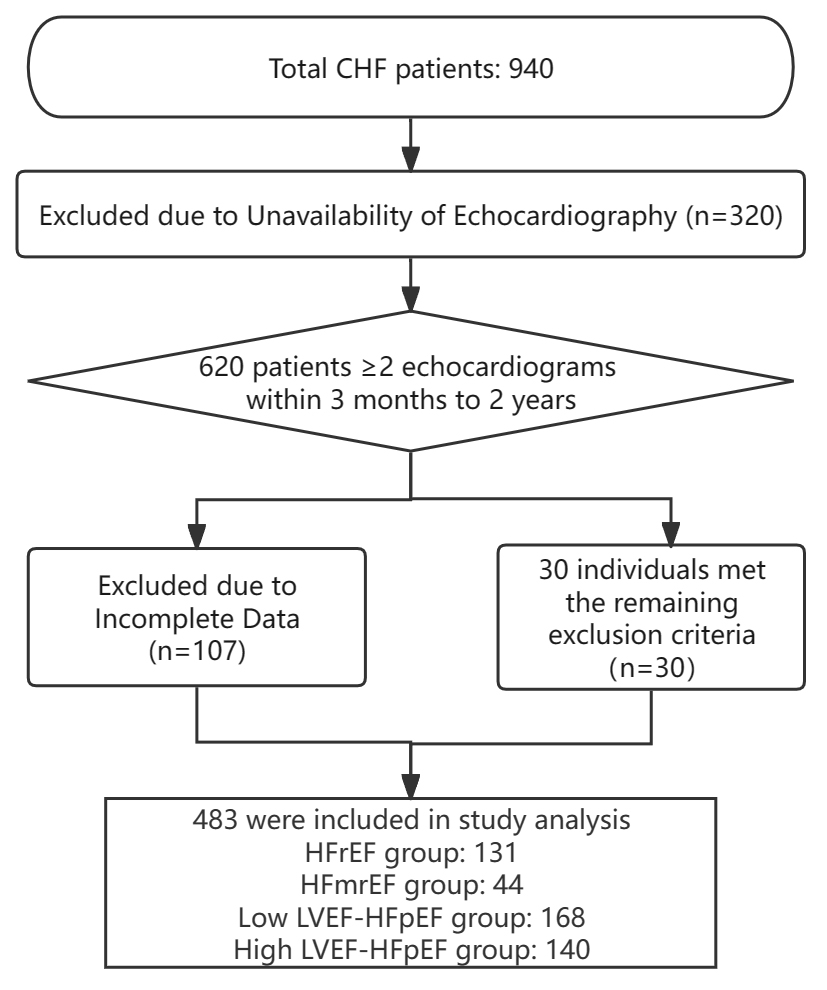
**Flow chart of the study**. CHF, chronic heart failure; HFrEF, HF with reduced ejection fraction; HFmrEF, HF with mildly reduced ejection fraction; LVEF, left ventricular ejection fraction; HFpEF, HF with preserved ejection fraction; HF, heart failure.

A total of 940 patients diagnosed with chronic HF were identified, and after applying the inclusion and exclusion criteria, 483 patients were ultimately included in the study. The mean age of the patients was 71.6 ± 13.0 years, and 60.0% were male. Among them, there were 131 patients in the HFrEF group, 44 in the HFmrEF group, 168 in the low LVEF-HFpEF group, and 140 in the high LVEF-HFpEF group (Fig. 1).

### 3.2 Baseline Characteristics

There were differences in several baseline characteristics among the four groups, including age, sex, systolic blood pressure, diastolic blood pressure, heart rate (HR), baseline left ventricular end-diastolic diameter (LVEDD), baseline left ventricular end-systolic diameter (LVESD), New York Heart Association (NYHA) class III/IV, atrial fibrillation (AF), hypertension, cerebrovascular disease, chronic obstructive pulmonary disease (COPD), hyperuricemia, anemia, angiotensin-converting enzyme inhibitor/angiotensin II receptor blocker/angiotensin receptor neprilysin inhibitor (ACEI/ARB/ARNI) usage, calcium channel blocker (CCB) usage, antiplatelet medication usage, digitalis usage, hemoglobin levels, and creatinine levels (Table 1).

**Table 1.** **Baseline clinical characteristics based on clinical phenotype of HF in HF patients**.

		N (%)				
		HFrEF	HFmrEF	Low LVEF-HFpEF	High LVEF-HFpEF	*p*-value
Characteristic		(n = 131, 27.1)	(n = 44, 9.1)	(n = 168, 34.8)	(n = 140, 29)	
	Age, years	66.0 ± 13.3	70.5 ± ${\mathrm{13.3}}^{a}$	74.0 ± ${\mathrm{11.8}}^{a}$	74.4 ± ${\mathrm{12.3}}^{a}$	<0.001
	Male	100 (76.3%)	32 (72.7%)	96 ${\mathrm{(57.1\%)}}^{a}$	62 ${\mathrm{(44.3\%)}}^{a\,b}$	<0.001
	BMI, ${\mathrm{kg/m}}^{2}$	24.2 (21.6, 27.3)	24.2 (21.5, 28.4)	24.3 (21.9, 27.3)	24.4 (22.2, 27.5)	0.732
	Systolic pressure, mmHg	117.7 ± 19.9	136.7 ± ${\mathrm{24.5}}^{a}$	141.0 ± ${\mathrm{22.5}}^{a}$	138.0 ± ${\mathrm{25.4}}^{a}$	<0.001
	Diastolic pressure, mmHg	76 (65, 85)	78.5 (72, 89.5)	79 ${\mathrm{(70, 89.8)}}^{d}$	74 ${\mathrm{(65.3, 84)}}^{c}$	0.022
	Heart rate, beats per min	87 (71, 100)	78.5 (71, 89.8)	79.5 ${\mathrm{(68, 93)}}^{a\,d}$	73.5 ${\mathrm{(65.3, 85)}}^{a\,c}$	<0.001
	Smoking	26 (19.8%)	9 (20.5%)	25 (14.9%)	23 (16.4%)	0.645
HF characteristics						
	Baseline LVEDD, mm	64 (58, 69)	56 ${\mathrm{(52, 60)}}^{a\,c\,d}$	49 ${\mathrm{(45, 54)}}^{a\,b}$	48 ${\mathrm{(44, 51.8)}}^{a\,b}$	<0.001
	Baseline LVESD, mm	55 (48, 6)	43 ${\mathrm{(39, 46.8)}}^{a\,c\,d}$	34 ${\mathrm{(30, 38)}}^{a\,b}$	31${\mathrm{(29, 35.8)}}^{a\,b}$	<0.001
	NYHA class III/IV	123 (93.9%)	35 ${\mathrm{(79.5\%)}}^{a}$	132 ${\mathrm{(78.6\%)}}^{a}$	111 ${\mathrm{(79.3\%)}}^{a}$	0.002
	${\mathrm{E/e}}^{’}$>15	80 (61.1%)	24 (54.5%)	74 ${\mathrm{(44.0\%)}}^{a}$	61 ${\mathrm{(43.6\%)}}^{a}$	0.010
Comorbidity						
	Atrial fibrillation	33 (25.2%)	10 (22.7%)	86 ${\mathrm{(51.2\%)}}^{a\,b}$	72 ${\mathrm{(51.4\%)}}^{a\,b}$	<0.001
	Hypertension	64 (48.9%)	35 ${\mathrm{(79.5\%)}}^{a}$	116 ${\mathrm{(69.0\%)}}^{a}$	108 ${\mathrm{(77.1\%)}}^{a}$	<0.001
	Coronary artery disease	94 (71.8%)	35 (79.5%)	117 (69.6%)	92 (65.7%)	0.343
	Valvular heart disease	44 (33.6%)	11 (25.0%)	69 (41.1%)	52 (37.1%)	0.209
	PCI	20 (15.3%)	8 (18.2%)	23 (13.7%)	14 (10.0%)	0.448
	CABG	4 (3.1%)	0 (0%)	7 (4.2%)	3 (2.1%)	0.463
	Cerebrovascular disease	37 (28.2%)	20 (45.4%)	80 ${\mathrm{(47.6\%)}}^{a}$	50 (35.7%)	0.005
	COPD	6 (4.6%)	2 (4.5%)	5 (3.0%)	15 ${\mathrm{(10.7\%)}}^{c}$	0.028
	Diabetes	46 (35.1%)	17 (38.6%)	75 (44.6%)	50 (35.7%)	0.293
	Chronic kidney disease	34 (26.0%)	16 (36.4%)	49 (29.2%)	32 (22.9%)	0.304
	Hyperlipidemia	23(17.6%)	9 (20.5%)	22 (13.1%)	27 (19.3%)	0.432
	Hyperuricemia	58 (44.3%)	15 (34.1%)	48 ${\mathrm{(28.6\%)}}^{a}$	26 ${\mathrm{(18.6\%)}}^{a}$	<0.001
	Hypoalbuminemia	24 (18.3%)	8 (18.2%)	44 (26.2%)	24 (17.1%)	0.188
	Anemia	19 (14.5%)	13 (29.5%)	53 ${\mathrm{(31.5\%)}}^{a}$	34 (24.3%)	0.007
	Pacemaker	3 (2.3%)	1 (2.3%)	9 (5.4%)	5 (3.6%)	0.519
	ICD	1 (0.8%)	0 (0%)	0 (0%)	1 (0.7%)	0.665
Therapy						
	ACEI/ARB/ARNI	82 (62.6%)	18 (40.9%)	73 ${\mathrm{(43.5\%)}}^{a}$	57 ${\mathrm{(40.7\%)}}^{a}$	0.001
	CCB	10 (7.6%)	14 ${\mathrm{(31.8\%)}}^{a}$	56 ${\mathrm{(33.3\%)}}^{a}$	54 ${\mathrm{(38.6\%)}}^{a}$	<0.001
	β-blocker	89 (68.0%)	29 (65.9%)	130 (77.4%)	88 ${\mathrm{(62.9\%)}}^{c}$	0.041
	Aldosterone antagonist	85 (64.9%)	25 (56.8%)	99 (58.9%)	87 (62.1%)	0.677
	Diuretics	102 (77.9%)	29 (65.9%)	118 (70.2%)	88 ${\mathrm{(62.9\%)}}^{a}$	0.056
	Antiplatelet agents	94 (71.8%)	31 (70.5%)	91 ${\mathrm{(54.2\%)}}^{a}$	74 ${\mathrm{(52.9\%)}}^{a}$	0.002
	Oral anticoagulations	15 (11.5%)	9 (20.5%)	38 (22.6%)	35 ${\mathrm{(25.0\%)}}^{a}$	0.031
	Statin	81 (61.8%)	28 (63.6%)	111 (66.1%)	97 (69.3%)	0.624
	Digitalis	37 (28.2%)	7 (15.9%)	8 ${\mathrm{(4.8\%)}}^{a}$	6 ${\mathrm{(4.3\%)}}^{a}$	<0.001
Laboratory data						
	Hemoglobin, g/L	134.6 ± 20.2	127.6 ± ${\mathrm{24.5}}^{c}$	115.64 ± ${\mathrm{26.7}}^{a\,b}$	119.59 ± ${\mathrm{23.8}}^{a}$	<0.001
	Potassium, mmol/L	4.1 (3.8, 4.4)	4.1 (3.8, 4.4)	4 (3.7, 4.5)	4 (3.5, 4.3)	0.155
	Creatinine, umol/L	94 (81.3, 119.9)	98.5 ${\mathrm{(78.6, 129.4)}}^{d}$	93.7 ${\mathrm{(73.7, 132.8)}}^{d}$	82.8 ${\mathrm{(67.9, 107.8)}}^{a\,b\,c}$	0.002
	CK-MB, U/L	14.1 (11.9, 19.2)	13.35 (11.6, 19.5)	14.1 (11.2, 18.5)	14.2 (11.925, 17.5)	0.809
	eGFR, mL/min/1.73/$m^{2}$	66.3 ± 24.5	61.9 ± ${\mathrm{26.5}}^{d}$	64.4 ± ${\mathrm{30.9}}^{d}$	72.8 ± ${\mathrm{31.6}}^{b\,c}$	0.041
	CRP, mg/L	7.24 (1.54, 24.08)	5.92 (1.48, 17.2425)	7.365 (2.2975, 23.57)	8.65 (2.82, 28.435)	0.604

Abbreviations: HF, heart failure; BMI, body mass index; LVEF, left ventricular ejection fraction; NYHA, New York Heart Association; LVEDD, left ventricular end-diastolic diameter; LVESD, left ventricular end-systolic diameter; eGFR, estimate glomerular filtration rate; PCI, percutaneous coronary intervention; CABG, coronary artery bypass graft; COPD, chronic obstructive pulmonary disease; ICD, implantable cardioverter-defibrillator; CCB, calcium channel blockers; CK-MB, creatine kinase MB; ACEI, angiotensin-converting enzyme inhibitors; ARB, angiotensin II receptor blockers; ARNI, angiotensin receptor-neprilysin inhibitors; CRP, C-reaction protein; HFmrEF, HF with mildly reduced ejection fraction; HFrEF, HF with reduced ejection fraction; HFpEF, HF with preserved ejection fraction. (1) a, *p* < 0.05 versus HFrEF; (2) b, *p* < 0.05 versus HFmrEF; (3) c, *p* < 0.05 versus Low LVEF-HFpEF; (4) d, *p* < 0.05 versus High LVEF-HFpEF.

Specifically, the low LVEF-HFpEF group differed significantly from the HFrEF group in terms of age, systolic blood pressure, HR, follow-up LVEF, baseline LVEDD, baseline LVESD, NYHA class III/IV, AF, hypertension, cerebrovascular disease, hyperuricemia, anemia, ACEI/ARB/ARNI usage, CCB usage, antiplatelet medication usage, digitalis usage, and hemoglobin levels (*p* < 0.05) (Table 1). When comparing the high LVEF-HFpEF group to the HFrEF group, significant differences were observed in age, sex, systolic blood pressure, HR, baseline LVEDD, baseline LVESD, NYHA class III/IV, AF, hypertension, hyperuricemia, ACEI/ARB/ARNI usage, CCB usage, β-blocker usage, aldosterone receptor antagonist usage, diuretic usage, antiplatelet medication usage, digitalis usage, hemoglobin levels, and creatinine levels (*p* < 0.05) (Table 1).

### 3.3 Kaplan‒Meier Curves for Overall and Cardiovascular Mortality

**Fig. 2.**
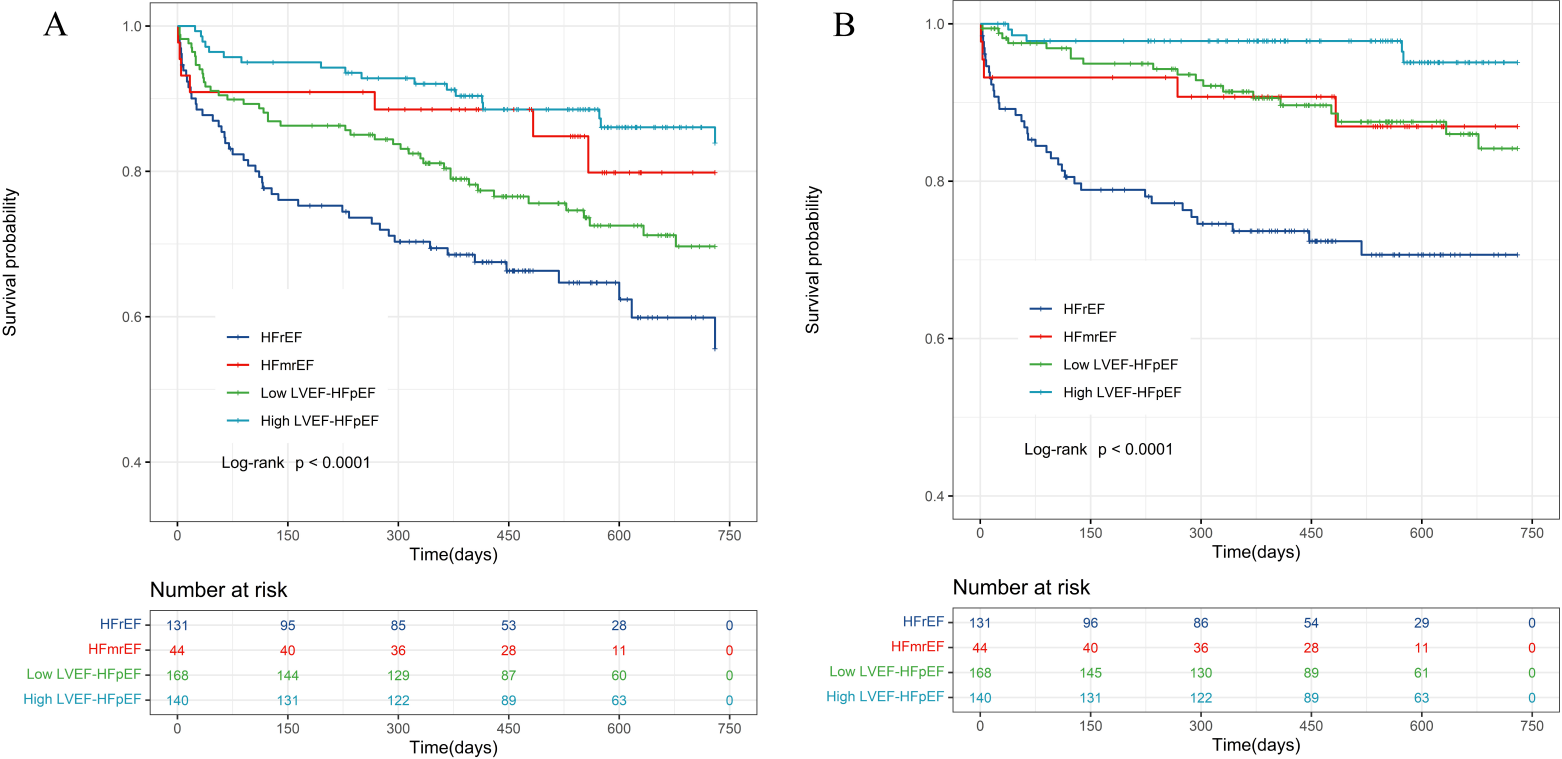
**Kaplan-Meier survival curve for all heart failure patients**. (A) Kaplan-Meier curves for the all-cause death of all HF phenotypes. (B) Kaplan-Meier curves for the cardiovascular death of all HF phenotypes. HF, heart failure; LVEF, left ventricular ejection fraction; HFmrEF, HF with mildly reduced ejection fraction; HFrEF, HF with reduced ejection fraction; HFpEF, HF with preserved ejection fraction.

**Fig. 3.**
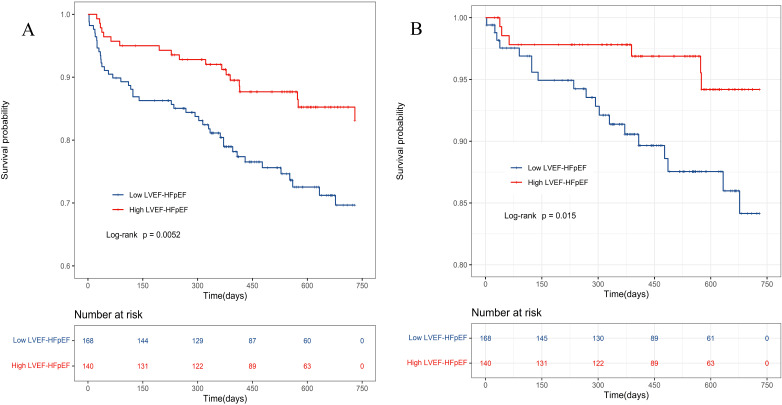
**Kaplan-Meier survival curve for HFpEF patients**. (A) Kaplan-Meier survival curves depicting the cumulative all-cause mortality rates of two groups of HFpEF patients. (B) Kaplan-Meier survival curves depicting the cumulative cardiovascular mortality rates of two groups of HFpEF patients. LVEF, left ventricular ejection fraction; HFpEF, heart failure with preserved ejection fraction.

During a median follow-up period of 414.0 (118.0, 569.0) days, a total of 114 patients (23.6%) experienced all-cause mortality, including 46 patients (9.5%) in the HFrEF group, 7 patients (1.4%) in the HFmrEF group, 43 patients (8.9%) in the low LVEF-HFpEF group, and 18 patients (3.7%) in the high LVEF-HFpEF group. The comparison of all-cause mortality rates among the four groups showed statistically significant differences (χ^2^ = 24.888, *p* < 0.001) (Fig. 2A). There were 64 patients (13.3%) who experienced cardiovascular mortality, including 35 patients (7.2%) in the HFrEF group, 5 patients (1.0%) in the HFmrEF group, 19 patients (3.9%) in the low LVEF-HFpEF group, and 5 patients (1.0%) in the high LVEF-HFpEF group. Comparison of cardiovascular mortality rates among the four groups also revealed statistically significant differences (χ^2^ = 38.03, *p* < 0.001) (Fig. 2B). Kaplan‒Meier curves were separately plotted for the low LVEF-HFpEF and high LVEF-HFpEF groups. Kaplan‒Meier analysis demonstrated statistically significant differences between these two groups in terms of overall and cardiovascular mortality (*p* < 0.05) (Fig. 3).

### 3.4 Analysis of Risk Factors for Endpoint Events

A multivariate Cox regression model was used to analyze the independent risk factors for all-cause mortality in all HF patients. The results showed that baseline LVEF, group classification, age, body mass index (BMI), estimated glomerular filtration rate (eGFR), and diuretic use were independent risk factors for all-cause mortality (*p* < 0.05) (**Supplementary Table 1**). In terms of cardiovascular mortality, multivariate Cox regression analysis indicated that age, HR, blood potassium levels, baseline LVEDD, and baseline LVESD were independent risk factors for cardiovascular mortality in all HF patients (*p* < 0.05) (**Supplementary Table 2**).

Separate multivariate Cox regression analyses were performed for the low LVEF-HFpEF group and the high LVEF-HFpEF group. In the low LVEF-HFpEF group, multivariate Cox regression analysis revealed that BMI, coronary artery disease, cerebrovascular disease, hyperlipidemia, hypoalbuminemia, and diuretic use were independent risk factors for all-cause mortality (*p* < 0.05, Table 2). Meanwhile, hyperlipidemia, eGFR, and β-blocker usage were identified as independent risk factors for cardiovascular mortality (*p* < 0.05, Table 3). In the high LVEF-HFpEF group, multivariate Cox regression analysis revealed that age, smoking history, hypoalbuminemia, and the eGFR were independent risk factors for all-cause mortality, while age, HR, blood potassium levels, and the eGFR were important independent risk factors for cardiovascular mortality (*p* < 0.05, Tables 4,5).

**Table 2.** **Univariate and multivariate Cox regression for all-cause mortality in the low LVEF-HFpEF group**.

	Univariate			Multivariate					
	HR	95% CI	*p*-value	β	SE	Wald$\chi^{2}$	HR	95% CI	*p*-value
Age, years	1.036	1.006–1.068	0.019	-	-	-	-	-	-
Male	1.114	0.604–2.053	0.730	-	-	-	-	-	-
BMI, ${\mathrm{kg/m}}^{2}$	0.851	0.780–0.930	<0.001	–0.150	0.049	9.513	0.861	0.783–0.947	0.002
Atrial fibrillation	1.693	0.917–3.125	0.092	-	-	-	-	-	-
Coronary artery disease	2.381	1.296–4.375	0.005	1.554	0.355	19.221	4.732	2.362–9.482	<0.001
Cerebrovascular disease	2.244	1.198–4.203	0.012	0.696	0.337	4.266	2.006	1.036–3.883	0.039
Hyperlipidemia	2.145	1.027–4.478	0.042	0.987	0.403	6.012	2.684	1.219–5.908	0.014
Hypoalbuminemia	0.307	0.169–0.559	<0.001	0.834	0.334	6.220	2.302	1.196–4.434	0.013
Baseline LVEDD	0.960	0.915–1.007	0.092	-	-	-	-	-	-
eGFR, mL/min/1.73/$m^{2}$	0.991	0.981–1.001	0.079	-	-	-	-	-	-
β-blocker	0.371	0.201–0.685	0.002	-	-	-	-	-	-
Diuretics	0.503	0.274–0.923	0.026	–1.290	0.351	13.527	0.275	0.138–0.547	<0.001
Antiplatelet agents	0.497	0.271–0.913	0.024	-	-	-	-	-	-
Oral anticoagulations	0.438	0.172–1.113	0.083	–1.257	0.492	6.525	0.285	0.108–0.746	0.011
Statin	0.542	0.296–0.993	0.048	-	-	-	-	-	-

Abbreviations: BMI, body mass index; LVEDD, left ventricular end-diastolic diameter; eGFR, estimate glomerular filtration rate; -, not applicable; HR, hazard ratio; CI, confidence interval; LVEF, left ventricular ejection fraction; HFpEF, heart failure with preserved ejection fraction; SE, standard error.

**Table 3.** **Univariate and multivariate Cox regression for cardiovascular disease mortality in the low LVEF-HFpEF group**.

	Univariate			Multivariate					
	HR	95% CI	*p*-value	β	SE	Wald$\chi^{2}$	HR	95% CI	*p*-value
Age, years	1.031	0.987–1.077	0.172	-	-	-	-	-	-
Male	1.205	0.474–3.065	0.695	-	-	-	-	-	-
BMI, ${\mathrm{kg/m}}^{2}$	0.878	0.773–0.998	0.046	-	-	-	-	-	-
Systolic pressure, mmHg	1.019	1.000–1.038	0.056	-	-	-	-	-	-
Hyperlipidemia	3.045	1.095–8.472	0.033	1.397	0.537	6.758	4.044	1.410–11.594	0.009
Hypoalbuminemia	2.382	0.958–5.923	0.062	-	-	-	-	-	-
eGFR, mL/min/1.73/$m^{2}$	0.983	0.967–0.998	0.029	–0.018	0.008	5.061	0.982	0.967–0.998	0.024
β-blocker	0.211	0.086–0.521	0.001	–1.582	0.468	11.436	0.206	0.082–0.514	0.001

Abbreviations: BMI, body mass index; eGFR, estimate glomerular filtration rate; -, not applicable; HR, hazard ratio; CI, confidence interval; LVEF, left ventricular ejection fraction; HFpEF, heart failure with preserved ejection fraction; SE, standard error.

**Table 4.** **Univariate and multivariate Cox regression for all-cause mortality in the high LVEF-HFpEF group**.

	Univariate			Multivariate					
	HR	95% CI	*p*-value	β	SE	Wald$\chi^{2}$	HR	95% CI	*p*-value
Age, years	1.090	1.030–1.155	0.003	0.116	0.029	15.601	1.123	1.060–1.190	<0.001
Male	1.860	0.721–4.799	0.200	-	-	-	-	-	-
COPD	2.592	0.849–7.916	0.094	-	-	-	-	-	-
Chronic kidney disease	2.267	0.878–5.854	0.091	-	-	-	-	-	-
Smoking	3.338	1.293–8.617	0.013	1.331	0.530	6.317	3.785	1.340–10.687	0.012
Hypoalbuminemia	2.741	1.026–7.320	0.044	1.911	0.579	10.884	6.758	2.172–21.030	0.001
Anemia	2.746	1.083–6.961	0.033	-	-	–	-	-	-
Baseline LVEDD	0.938	0.879–1.002	0.057	-	-	-	-	-	-
Baseline LVESD	0.892	0.808–0.985	0.024	-	-	-	-	-	-
Hemoglobin	0.983	0.964–1.002	0.076	-	-	-	-	-	-
eGFR, mL/min/1.73/$m^{2}$	0.982	0.967–0.997	0.021	–0.018	0.009	3.952	0.982	0.964–1.000	0.047
Oral anticoagulations	0.173	0.023–1.300	0.088	-	-	-	-	-	-

Abbreviations: LVEDD, left ventricular end-diastolic diameter; LVESD, left ventricular end-systolic diameter; eGFR, estimate glomerular filtration rate; -, not applicable; HR, hazard ratio; CI, confidence interval; LVEF, left ventricular ejection fraction; HFpEF, heart failure with preserved ejection fraction; SE, standard error.

**Table 5.** **Univariate and multivariate Cox Regression for cardiovascular disease mortality in the high LVEF-HFpEF group**.

	Univariate			Multivariate					
	HR	95% CI	*p*-value	β	SE	Wald$\chi^{2}$	HR	95% CI	*p*-value
Age, years	1.218	1.052–1.411	0.008	0.225	0.102	4.877	1.252	1.026–1.529	0.027
Male	1.758	0.293–10.530	0.537	-	-	-	-	-	-
Heart rate, beats per min	1.050	1.004–1.097	0.034	-	-	-	-	-	-
Hyperuricemia	6.267	1.044–37.619	0.045	-	-	-	-	-	-
Potassium, mmol/L	2.161	1.120–4.168	0.022	1.195	0.429	7.762	3.304	1.425–7.66	0.005
eGFR, mL/min/1.73/$m^{2}$	0.960	0.928–0.994	0.020	-	-	-	-	-	-

Abbreviations: eGFR, estimate glomerular filtration rate; -, not applicable; HR, hazard ratio; CI, confidence interval; LVEF, left ventricular ejection fraction; HFpEF, heart failure with preserved ejection fraction; SE, standard error.

### 3.5 Sensitivity Analysis

**Fig. 4.**
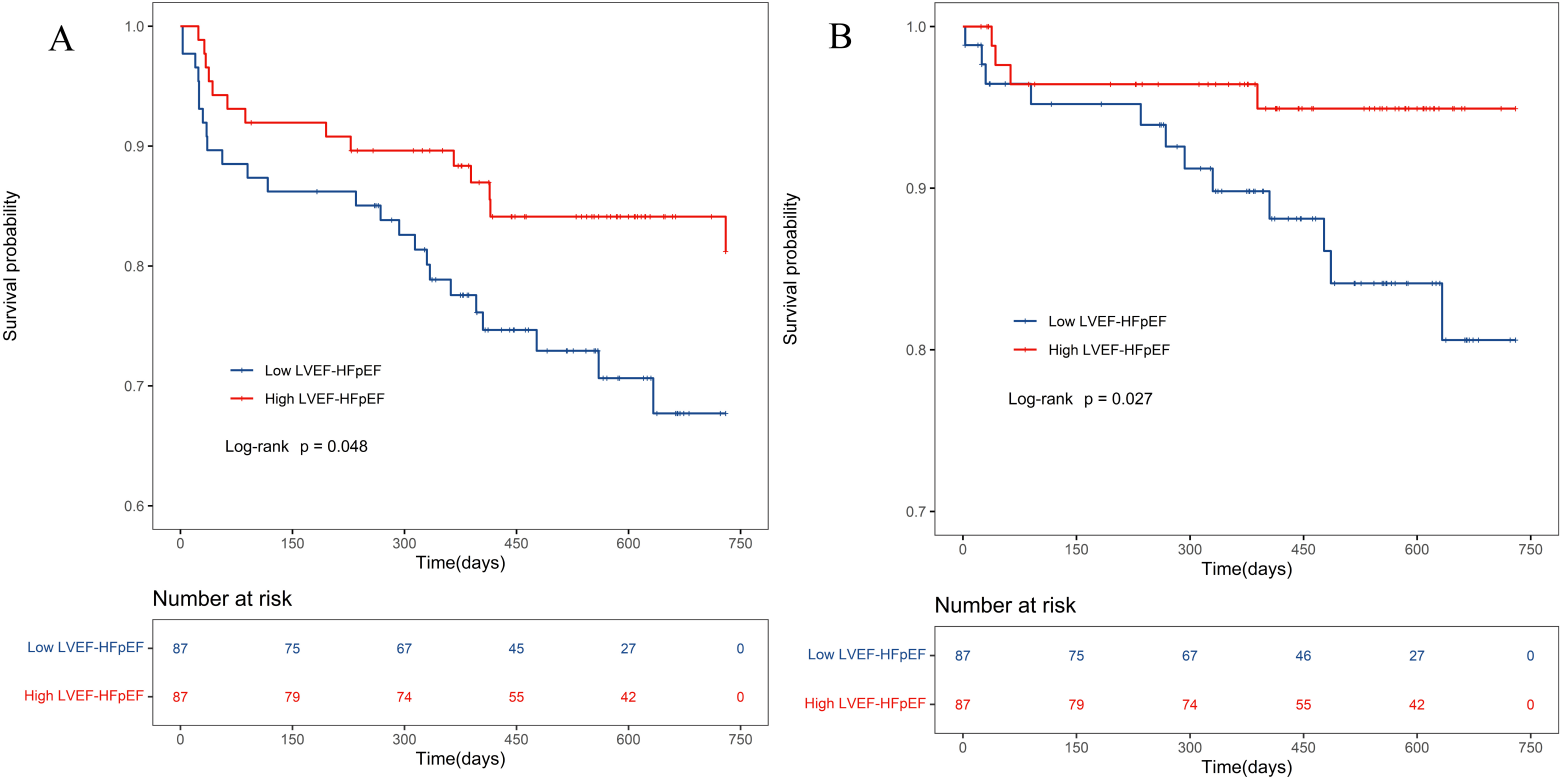
**Kaplan-Meier survival curve of HFpEF patients after PSM**. (A) All-cause death in the PSM population. (B) Cardiovascular death in the PSM population. PSM, propensity score matching. LVEF, left ventricular ejection fraction; HFpEF, heart failure with preserved ejection fraction.

Comparability between the low LVEF-HFpEF and high LVEF-HFpEF groups was adjusted using 1:1 PSM. Baseline indicators between the two groups were included in the analysis, with a caliper value set at 0.2. A total of 87 pairs were successfully matched, and after PSM, there were no statistically significant differences in baseline data between the two groups (*p* > 0.05) (**Supplementary Table 3**). The low LVEF-HFpEF group had higher rates of all-cause mortality (*p* = 0.048) (Fig. 4A) and cardiovascular mortality (*p* = 0.027) (Fig. 4B) than the high LVEF-HFpEF group.

### 3.6 Competing Risk Analysis

**Fig. 5.**
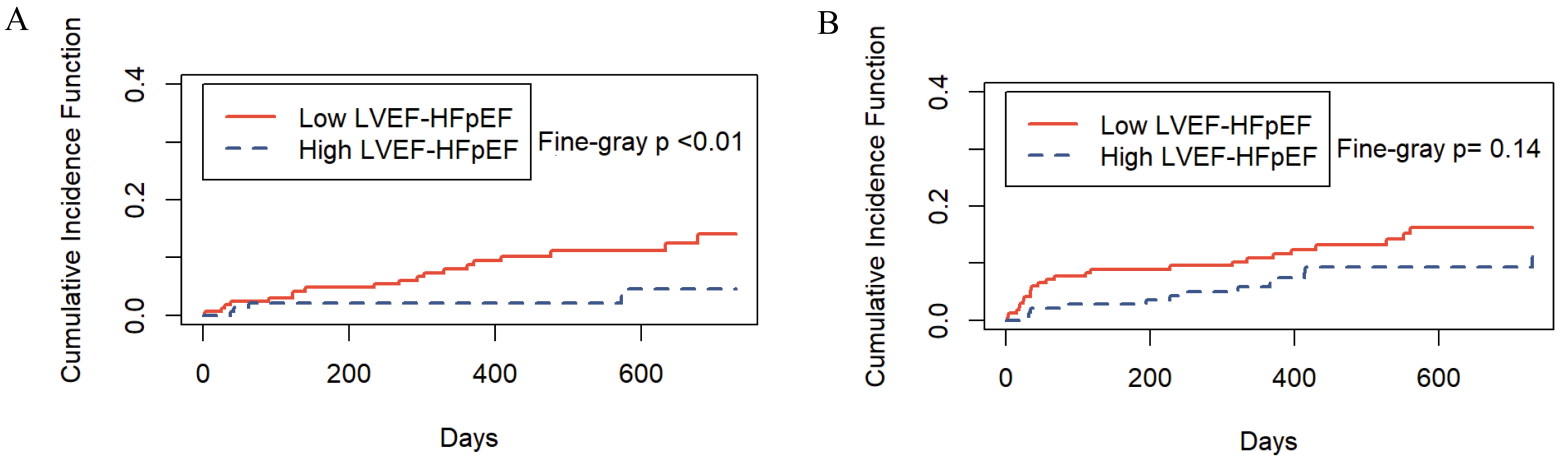
**Incidence of cardiovascular and noncardiovascular deaths by HFpEF phenotype**. (A) The risk of cardiovascular death. (B) The risk of competing events. LVEF, left ventricular ejection fraction; HFpEF, heart failure with preserved ejection fraction.

During the follow-up period, differences were observed in cardiovascular and noncardiovascular deaths among different HFpEF phenotypes. Therefore, noncardiovascular death was considered a competing event in the competing risk analysis. The results showed that the cardiovascular mortality risk of the high LVEF-HFpEF group was lower than that of the low LVEF-HFpEF group (Fine-Gray *p* < 0.01) (Fig. 5A). However, there was no statistically significant difference in the risk of competing events between the two groups (Fine-Gray *p* = 0.14) (Fig. 5B).

## 4. Discussion

While there has been a considerable amount of research investigating the clinical characteristics and outcomes of HFpEF, available data on HFpEF subtypes based on different LVEF ranges are limited. This is partly due to the categorization of HF patients with LVEF >50% as a single independent phenotype, which has, to some extent, restricted further observations of potential subtypes.

The focus of this study was to explore the clinical features and prognosis of HFpEF within different LVEF ranges. This study revealed significant differences in clinical characteristics and two-year survival rates among HFpEF subtypes with varying LVEF ranges. Compared to the high LVEF-HFpEF group, the low LVEF-HFpEF group exhibited higher diastolic blood pressure and faster HR, suggesting that there may be an underlying decrease in arterial elasticity and myocardial contractility in patients in the low LVEF-HFpEF group.

In the past, HFpEF was traditionally considered to be solely caused by diastolic dysfunction of the heart. However, recent research has revealed that HFpEF also encompasses significant impairment in systolic function and a restricted contractile reserve during periods of stress [7, 8]. When cardiac contractility declines, LVEF decreases, and the heart needs to beat faster to maintain adequate blood supply. Therefore, patients with lower LVEF may exhibit a higher HR as the disease progresses normally.

Furthermore, in comparison to the high LVEF-HFpEF group, patients in the low LVEF-HFpEF group had higher levels of serum creatinine and lower eGFR. Notably, there were no significant differences between the low LVEF-HFpEF group and the HFmrEF or HFrEF groups. This suggests that low LVEF-HFpEF may share similarities with HFmrEF and HFrEF. In this study, for each unit increase in eGFR, there was a 1.2% reduction in the risk of all-cause mortality and a 1.8% reduction in the risk of cardiovascular mortality among HF patients (Tables 2,3). The findings indicate a negative correlation between the eGFR and both all-cause and cardiovascular mortality in HF, which aligns with previous research [9, 10, 11]. This correlation may be related to improved renal perfusion at higher LVEF levels. The intricate relationship between the heart and kidneys has long been a subject of extensive research [12], involving mechanisms such as neurohumoral drive, autonomic reflexes, and fluid balance alterations, all of which collectively maintain circulatory and internal environmental homeostasis. However, the findings of this study underscore the differences in HFpEF subtypes within different LVEF ranges, possibly reflecting distinct pathological processes between these two HFpEF subtypes.

This study observed differences in survival rates among various HF phenotypes, with the high LVEF-HFpEF group having the lowest mortality rate and the HFrEF group having the highest. In contrast, the survival rates of the low LVEF-HFpEF and HFmrEF groups were relatively close. HFmrEF patients are typically situated on a dynamic trajectory transitioning from HFrEF to either improvement or deterioration [6, 13]. This suggests that low LVEF-HFpEF may also represent a clinically unstable phenotype. Therefore, dynamic monitoring of LVEF changes over time and actively searching for the causes of EF variation are crucial.

After PSM, the survival rate of the low LVEF-HFpEF group remained lower than that of the high LVEF-HFpEF group (Fig. 4A,B). This difference may be attributed to variations in cardiac morphology and pathophysiology between the two subtypes. Sebastian Rosch and colleagues analyzed patients with HFpEF whose LVEF fell within the ranges of 50%–60% and LVEF >60% using various diagnostic methods, including imaging (including echocardiography and cardiac magnetic resonance imaging), histology (myocardial biopsy), and hemodynamic catheterization (conduction catheter). Their findings indicated that HFpEF patients with LVEF in the range of 50%–60% exhibited reduced contractility, impaired ventricular-arterial coupling, and increased myocardial fibrosis. In contrast, patients with LVEF >60% exhibited a high-systolic state characterized by left ventricular afterload excess and reduced preload reserve, suggesting fundamental differences in cardiac morphology and hemodynamic responses between high LVEF-HFpEF and low LVEF-HFpEF [5]. This, to some extent, explains the varying prognoses observed in HFpEF patients with different LVEF ranges. Furthermore, even after accounting for competing risks, there still existed a difference in cardiovascular mortality between the two groups (Fig. 5A), consistent with the findings of Gu *et al*. [14]. However, it should be noted that with regard to noncardiovascular death, there was no significant difference between the high LVEF-HFpEF and low LVEF-HFpEF groups (Fig. 5B). This suggests that the two groups share some degree of homogeneity in certain aspects.

This study showed that HF patients with hypoalbuminemia were more likely to experience adverse outcomes. Hypoalbuminemia leads to decreased plasma oncotic pressure and effective circulating blood volume, exacerbating microcirculation and multiorgan dysfunction. Additionally, reduced serum albumin levels diminish antibody production and immune function while increasing the risk of various infections, further deteriorating the condition of HF patients [15]. Furthermore, elevated potassium (K+) levels were associated with an increased cardiovascular disease (CVD) risk in high LVEF-HFpEF patients. Blood potassium levels were reported to be independently associated with hospitalization and long-term mortality events in HF patients, exhibiting a “U”-shaped curve pattern [16]. Moreover, HF patients possess multiple risk factors for hyperkalemia, including advanced age, diabetes, chronic kidney disease, or metabolic acidosis, all of which are associated with an increased risk of hospitalization and mortality [17]. Therefore, the importance of monitoring blood potassium levels should not be underestimated in the management of HF patients.

This study did not reveal any significant impact of ACEI, ARB, or ARNI on the prognosis of HFpEF. Additionally, due to the timeframe of the study, sodium-glucose cotransporter-2 inhibitors (SGLT2is) were not included in this research study. Initially, recommended for treating HFrEF, recent studies have suggested that SGLT2is exhibit significant therapeutic potential for HF patients across all LVEF categories, in part due to their capacity to reduce blood volume in HF patients [18, 19]. Reducing blood volume can alleviate cardiac load, improve both systolic and diastolic function, decrease myocardial oxygen consumption, and potentially enhance cardiac structure and function. Hence, it is a valuable strategy for HF patients. However, HFpEF patients exhibit notable differences in hemodynamics and myocardial fibrosis between resting and exercise states, with patients having LVEF >60% showing reduced ventricular size and increased diastolic and systolic stiffness, potentially limiting their response to volume regulation [5]. This observation nicely explains the varying effects of diuretics on the prognosis of different HFpEF subtypes in this study, i.e., diuretics can reduce the risk of endpoint events in HFpEF patients with lower LVEF, while this effect is not observed in patients with higher LVEF values. Hence, reducing blood volume may not be the optimal approach when treating HFpEF patients with higher LVEF.

Furthermore, this study revealed that β-blockers can lower cardiovascular mortality in low LVEF-HFpEF patients, which differs partially from previous research conclusions. In most clinical trials targeting HFrEF patients, β-blockers have shown positive effects, extending patient survival and reducing overall mortality, cardiovascular mortality, and the incidence of HF readmissions [20, 21, 22, 23]. However, in HFpEF, β-blockers have not shown significant benefits and may even be harmful [24, 25]. This disparity may be due to the distinct pathological characteristics of HFpEF patients compared to HFrEF, including various factors such as ventricular diastolic and systolic reserve function, HR reserve and rhythm, atrial dysfunction, ventricular and vascular stiffness, impaired vasodilation, pulmonary artery hypertension, endothelial dysfunction, and various other complex interactions involving peripheral tissues such as skeletal muscle. These mechanisms vary in their degree of involvement in the development of HFpEF, making the treatment of HFpEF more challenging [8]. One reason for these differences might be the exclusion of patients who did not undergo follow-up echocardiography in this study, which introduced some selection bias. Moreover, the partial similarity between low LVEF-HFpEF and HFmrEF/HFrEF could also contribute to these findings. Nevertheless, the results of this study suggest that there are potential subtypes within HFpEF, and investigating the efficacy of existing drugs for different HFpEF subtypes might be a future avenue of research. Therefore, a deeper exploration of the characteristics of HFpEF subtypes and tailored treatment strategies for different subtypes may be a focus of future research.

In summary, recognizing the clinical significance of observed differences among HFpEF subgroups, our study underscores the heterogeneity of the HFpEF population and the necessity for tailored therapeutic strategies. The distinct clinical characteristics and prognoses of low LVEF-HFpEF versus high LVEF-HFpEF subgroups suggest that a one-size-fits-all approach is insufficient for managing HFpEF patients. The low LVEF-HFpEF subgroup may benefit from more aggressive management of risk factors. Optimal management of comorbidities such as hyperlipidemia, coronary artery disease, and cerebrovascular disease, alongside vigilant monitoring of renal function and cardiac structural changes (i.e., LVEDD and LVESD), could be key to improving prognosis in this subgroup. Moreover, attention to nutritional status to ensure appropriate body mass index and improvement of hypoalbuminemia would be beneficial. Conversely, the high LVEF-HFpEF subgroup may benefit more from primary prevention strategies and lifestyle modifications. This includes smoking cessation programs and targeted dietary interventions such as appropriate potassium intake.

## 5. Limitations

(1) This is a single-center retrospective study. Although patients were rigorously selected based on inclusion and exclusion criteria, larger sample sizes and longer-term follow-up results are needed in the future to validate these findings. (2) A significant portion of HF patients in this study had received interventions at other health care institutions before their visits to the study center. For these patients, we did not have information on their EF before the intervention, and we did not obtain other information about them at that time, which could result in missing data bias. (3) This study primarily utilized noninvasive echocardiography to assess the LVEF in heart failure patients. Due to technical and resource limitations, we encountered challenges in collecting global longitudinal strain (GLS) data. Future research will aim to address these limitations to provide more comprehensive insights.

## 6. Conclusions

In conclusion, the results of this study support the notion that HFpEF with LVEF >60% and HFpEF with LVEF between 50% and 60% represent two distinct phenotypes. Patients with 50% ≤ LVEF ≤ 60% had a mortality rate similar to that of HFmrEF and better than that of HFrEF patients, suggesting that aggressive treatment of valvular heart disease and improvement in renal function may improve short-term prognosis and reduce mortality. On the other hand, for patients with LVEF >60%, diuretic therapy may not be the optimal treatment approach. In the future, practicing clinicians need to pay more attention to the differences between subtypes of HFpEF patients, especially in the selection of treatment strategies. We advocate for a stratified therapeutic approach to HFpEF patients tailored to the distinct clinical characteristics and risk profiles of different subtypes. A deeper understanding of the diversity within HFpEF subgroups is essential for developing more informed, precise, and effective management strategies.

## Data Availability

The datasets used and/or analyzed during the current study are available from the corresponding author on reasonable request.

## Supplementary Material

Supplementary material associated with this article can be found, in the online version, at https://doi.org/10.31083/j.rcm2505177.
