# Supplementary material for: Clinical Characteristics and Prognosis of Heart Failure with Preserved Ejection Fraction Across Diverse Ejection Fraction Ranges
Source: Rev Cardiovasc Med. 2024 May 20;25(5):177. doi: 10.31083/j.rcm2505177 (PMC11267200; doi:10.31083/j.rcm2505177)
Supplement: Supplementary file 1 [file 2153-8174-25-5-177-s1.docx]

Supplementary Table 1. univariate and multivariate Cox proportional hazards regression analysis of four groups of patients for all-cause mortality.

|  | univariate | | |  | multivariate | | | | | |
| --- | --- | --- | --- | --- | --- | --- | --- | --- | --- | --- |
|  | HR | 95%CI | p-value |  | β | SE | Waldχ^2^ | HR | 95%CI | p-value |
| Baseline LVEF,% | 0.974 | 0.962-0.985 | <0.001 |  | -0.046 | 0.017 | 7.212 | 0.955 | 0.923-0.988 | 0.007 |
| HFrEF |  |  | <0.001 |  |  |  | 9.831 |  |  | 0.015 |
| HFmrEF | 0.392 | 0.177-0.868 | 0.021 |  | -0.47 | 0.475 | 0.979 | 0.625 | 0.247-1.585 | 0.322 |
| Low LVEF-HFpEF | 0.624 | 0.412-0.947 | 0.027 |  | 0.664 | 0.522 | 1.62 | 1.943 | 0.699-5.403 | 0.203 |
| High LVEF-HFpEF | 0.282 | 0.164-0.488 | <0.001 |  | 0.033 | 0.604 | 0.003 | 1.034 | 0.316-3.379 | 0.956 |
| Age, years | 1.034 | 1.017-1.051 | <0.001 |  | 0.033 | 0.009 | 12.397 | 1.033 | 1.015-1.052 | <0.001 |
| Male | 1.503 | 1.01-2.238 | 0.045 |  | - | - | - | - | - | - |
| BMI, kg/m2 | 0.878 | 0.838-0.92 | <0.001 |  | -0.09 | 0.023 | 14.623 | 0.917 | 0.878-0.959 | <0.001 |
| Pulse,beats per min | 1.007 | 0.999-1.016 | 0.096 |  | - | - | - | - | - | - |
| Valvular heart disease | 1.538 | 1.064-2.224 | 0.022 |  | - | - | - | - | - | - |
| PCI | 0.56 | 0.293-1.072 | 0.08 |  | - | - | - | - | - | - |
| Cerebrovascular disease | 1.419 | 0.982-2.05 | 0.063 |  | - | - | - | - | - | - |
| Hyperuricemia | 1.474 | 1.009-2.152 | 0.045 |  | - | - | - | - | - | - |
| Hypoproteinemia | 2.301 | 1.562-3.391 | <0.001 |  | - | - | - | - | - | - |
| Anemia | 1.782 | 1.213-2.619 | 0.003 |  | - | - | - | - | - | - |
| NYHA class III/IV | 2.808 | 1.369-5.761 | 0.005 |  | - | - | - | - | - | - |
| Baseline LVESD, mm | 1.015 | 1-1.03 | 0.056 |  | - | - | - | - | - | - |
| Hemoglobin, g/L | 0.992 | 0.985-0.999 | 0.03 |  | - | - | - | - | - | - |
| Creatinine, umol/L | 1.002 | 1-1.004 | 0.049 |  | - | - | - | - | - | - |
| eGFR, mL⋅min^−1^⋅1.73 m^−2^ | 0.985 | 0.978-0.991 | <0.001 |  | -0.01 | 0.004 | 11.262 | 0.988 | 0.981-0.995 | 0.001 |
| Chronic kidney disease | 1.699 | 1.163-2.481 | 0.006 |  | - | - | - | - | - | - |
| ACEI/ARB/ARNI | 0.706 | 0.485-1.026 | 0.068 |  | - | - | - | - | - | - |
| CCB | 0.644 | 0.413-1.004 | 0.052 |  | - | - | - | - | - | - |
| β-blocker | 0.553 | 0.381-0.803 | 0.002 |  | - | - | - | - | - | - |
| Diuretics | 0.694 | 0.474-1.016 | 0.06 |  | -0.52 | 0.201 | 6.716 | 0.595 | 0.401-0.881 | 0.01 |
| Oral anticoagulations | 0.441 | 0.243-0.803 | 0.007 |  | -0.72 | 0.309 | 5.379 | 0.489 | 0.267-0.895 | 0.02 |
| Statin | 0.697 | 0.48-1.013 | 0.058 |  | - | - | - | - | - | - |

Abbreviations: HFrEF, heart failure with reduced ejection fraction; HFmrEF, heart failure with mid-range ejection fraction; HFpEF, heart failure with preserved ejection fraction; BMI, body mass index; LVEF, left ventricular ejection fraction; NYHA, New York Heart Association; LVESD, left ventricular end-systolic diameter; eGFR, estimate glomerular filtration rate; PCI, percutaneous coronary intervention; CCB, calcium channel blockers; ACEI, angiotensin-converting enzyme inhibitors; ARB, angiotensin II receptor blockers; ARNI, angiotensin receptor-neprilysin inhibitors. -, not applicable. HR, hazard ratio; CI, confidence interval;

Supplementary Table 2. univariate and multivariate Cox proportional hazards regression analysis of four groups of patients for cardiovascular mortality.

|  | univariate | | | |  | multivariate | | | | | | | | |
| --- | --- | --- | --- | --- | --- | --- | --- | --- | --- | --- | --- | --- | --- | --- |
|  | | HR | 95%CI | p-value | | |  | β | SE | Waldχ^2^ | | HR | 95%CI | p-value |
| Baseline LVEF,% | | 0.953 | 0.937-0.968 | <0.001 | | |  | -0.06 | 0.009 | | 43.699 | 0.939 | 0.922-0.957 | <0.001 |
| HFrEF | |  |  | <0.001 | | |  | - | - | | - | - | - | - |
| HFmrEF | | 0.37 | 0.145-0.945 | 0.038 | | |  | - | - | | - | - | - | - |
| Low LVEF-HFpEF | | 0.365 | 0.209-0.639 | <0.001 | | |  | - | - | | - | - | - | - |
| High LVEF-HFpEF | | 0.106 | 0.041-0.27 | <0.001 | | |  | - | - | | - | - | - | - |
| Age, years | | 1.022 | 1-1.043 | 0.045 | | |  | 0.032 | 0.011 | | 7.875 | 1.033 | 1.01-1.056 | 0.005 |
| Male | | 1.703 | 0.988-2.937 | 0.056 | | |  | - | - | | - | - | - | - |
| BMI, kg/m^2^ | | 0.893 | 0.84-0.95 | <0.001 | | |  | - | - | | - | - | - | - |
| Systolic pressure, mmHg | | 0.991 | 0.98-1.001 | 0.078 | | |  | - | - | | - | - | - | - |
| Valvular heart disease | | 1.724 | 1.056-2.816 | 0.029 | | |  | 0.532 | 0.254 | | 4.401 | 1.703 | 1.036-2.8 | 0.036 |
| Chronic kidney disease | | 2.187 | 1.335-3.584 | 0.002 | | |  | - | - | | - | - | - | - |
| Hyperuricemia | | 1.901 | 1.16-3.116 | 0.011 | | |  | - | - | | - | - | - | - |
| NYHA class III/IV | | 6.57 | 1.607-26.86 | 0.009 | | |  | - | - | | - | - | - | - |
| Baseline LVEDD, mm | | 1.039 | 1.018-1.06 | <0.001 | | |  | - | - | | - | - | - | - |
| Baseline LVESD, mm | | 1.042 | 1.024-1.061 | <0.001 | | |  | - | - | | - | - | - | - |
| Creatinine, umol/L | | 1.003 | 1.001-1.006 | 0.007 | | |  | - | - | | - | - | - | - |
| eGFR,mL⋅min^−1^⋅1.73 m^−2^ | | 0.98 | 0.971-0.989 | <0.001 | | |  | -0.02 | 0.005 | | 11.436 | 0.982 | 0.972-0.992 | 0.001 |
| CCB | | 0.484 | 0.253-0.927 | 0.029 | | |  | - | - | | - | - | - | - |
| β-blocker | | 0.47 | 0.287-0.77 | 0.003 | | |  | -0.61 | 0.256 | | 5.6 | 0.545 | 0.33-0.901 | 0.018 |
| Oral anticoagulations | | 0.46 | 0.21-1.008 | 0.052 | | |  | - | - | | - | - | - | - |
| Digitalis | | 1.727 | 0.922-3.236 | 0.088 | | |  | - | - | | - | - | - | - |

Abbreviations: HFrEF, heart failure with reduced ejection fraction; HFmrEF, heart failure with mid-range ejection fraction; HFpEF, heart failure with preserved ejection fraction; BMI, body mass index; LVEF, left ventricular ejection fraction; NYHA, New York Heart Association; LVEDD, left ventricular end-diastolic diameter; LVESD, left ventricular end-systolic diameter; eGFR, estimate glomerular filtration rate; CCB, calcium channel blockers. -, not applicable. HR, hazard ratio; CI, confidence interval;

Supplementary Table 3. Baseline Table after Propensity Score Matching (PSM).

|  | Low LVEF-HFpEF |  | High LVEF-HFpEF | |  | | p-value | |  |  |
| --- | --- | --- | --- | --- | --- | --- | --- | --- | --- | --- |
| Characteristic | (n=87, 50%) |  | (n=87, 50%) |  | |  |  | |  |  |
| Age, years | 73.8±11.3 |  | 73.4±11.8 |  | |  | 0.668 | |  |  |
| Male | 44 (50.6%) |  | 45 (51.7%) |  | |  | 0.879 | |  |  |
| BMI, kg/m^2^ | 23.95(21.7,27.1) |  | 25.12(22.9,27.7) |  | |  | 0.158 | |  |  |
| Systolic pressure, mmHg | 140.6±21.7 |  | 138.7±25.8 |  | |  | 0.601 | |  |  |
| Diastolic pressure, mmHg | 78.2±14.2 |  | 78.1±14.3 |  | |  | 0.966 | |  |  |
| Heart rate, beats per min | 81(66,95) |  | 77(66,89) |  | |  | 0.364 | |  |  |
| Smoking | 12 (13.8%) |  | 15 (17.2%) |  | |  | 0.530 | |  |  |
| HF characteristics |  |  |  |  | |  |  | |  |  |
| Baseline LVEF,% | 59(56,65) |  | 61(56,66) |  | |  | 0.356 | |  |  |
| Baseline LVEDD, mm | 49(44,54) |  | 49(45,53) |  | |  | 0.889 | |  |  |
| Baseline LVESD, mm | 33(30,38) |  | 32(30,38) |  | |  | 0.842 | |  |  |
| NYHA class III/IV | 69(79.3%) |  | 72(82.8%) |  | |  | 0.562 | |  |  |
| Comorbidity |  |  |  |  | |  |  | |  |  |
| Atrial fibrillation | 43(49.4%) |  | 45(51.7%) |  | |  | 0.762 | |  |  |
| Hypertension | 63(72.4%) |  | 64(73.6%) |  | |  | 0.864 | |  |  |
| coronary artery disease | 61(70.1%) |  | 63(72.4%) |  | |  | 0.738 | |  |  |
| Valvular heart disease | 36(41.4%) |  | 35(40.2%) |  | |  | 0.877 | |  |  |
| PCI | 13(14.9%) |  | 11(12.6%) |  | |  | 0.660 | |  |  |
| CABG | 2(2.3%) |  | 3(3.4%) |  | |  | 1 | |  |  |
| Cerebrovascular disease | 27(31.0%) |  | 34(39.1%) |  | |  | 0.266 | |  |  |
| COPD | 5(5.7%) |  | 3(3.4%) |  | |  | 0.717 | |  |  |
| Diabetes | 34(39.1%) |  | 31(35.6%) |  | |  | 0.638 | |  |  |
| Chronic kidney disease | 21(24.1%) |  | 18(20.7%) |  | |  | 0.585 | |  |  |
| Hyperlipidemia | 16(18.4%) |  | 15(17.2%) |  | |  | 0.843 | |  |  |
| Hyperuricemia | 19(21.8%) |  | 19(21.8%) |  | |  | 1 | |  |  |
| Hypoalbuminemia | 18(20.7%) |  | 21(24.1%) |  | |  | 0.585 | |  |  |
| Anemia | 23(26.4%) |  | 23(26.4%) |  | |  | 1 | |  |  |
| Pacemaker | 1(1.1%) |  | 1(1.1%) |  | |  | 1 | |  |  |
| Therapy |  |  |  |  | |  |  | |  |  |
| ACEI/ARB/ARNI | 36(41.4%) |  | 37(42.5%) |  | |  | 0.878 | |  |  |
| CCB | 28(32.2%) |  | 23(26.4%) |  | |  | 0.405 | |  |  |
| β-blocker | 62(71.3%) |  | 65(74.7%) |  | |  | 0.609 | |  |  |
| Aldosterone antagonist | 55(63.2%) |  | 56(64.4%) |  | |  | 0.875 | |  |  |
| Diuretics | 61(70.1%) |  | 61(70.1%) |  | |  | 1 | |  |  |
| Antiplatelet agents | 51(58.6%) |  | 48(55.2%) |  | |  | 0.646 | |  |  |
| Oral anticoagulations | 19(21.8%) |  | 19(21.8%) |  | |  | 1 | |  |  |
| Statin | 62(71.3%) |  | 62(71.3%) |  | |  | 1 | |  |  |
| Digitalis | 4(4.6%) |  | 5(5.7%) | | | | 1 |  | |  |
| Laboratory data |  |  |  | | | |  |  | |  |
| Hemoglobin, g/L | 123(102,140) |  | 119(103,137) | | | | 0.964 |  | |  |
| Potassium, mmol/L | 3.9(3.6,4.5) |  | 4(3.5,4.3) | | | | 0.706 |  | |  |
| Creatinine, umol/L | 83.6(67.3,114.9) |  | 85.6(69,110.3) | | | | 0.903 |  | |  |
| CK-MB, U/L | 14.1(11.2,18.8) |  | 14.1(12,16.8) | | | | 0.883 |  | |  |
| eGFR, mL/min/1.73/m^2^ | 71.3±30.3 |  | 74.5±31.4 | | | | 0.494 |  | |  |

Abbreviations: BMI, body mass index; LVEF, left ventricular ejection fraction; NYHA, New York Heart Association; LVEDD, left ventricular end-diastolic diameter; LVESD, left ventricular end-systolic diameter; eGFR, estimate glomerular filtration rate; PCI, percutaneous coronary intervention; CABG, coronary artery bypass graft;COPD, chronic obstructive pulmonary disease ;ICD, implantable cardioverter-defibrillator; CCB, calcium channel blockers; CK-MB, creatine kinase MB; ACEI, angiotensin-converting enzyme inhibitors; ARB, angiotensin II receptor blockers; ARNI, angiotensin receptor-neprilysin inhibitors. -, not applicable.
